# A novel enrichment-free, low-volume filtration and rapid lysis (ELR) method in combination with real-time PCR for detection of Shiga toxin-producing Escherichia coli (STEC) in water

**DOI:** 10.1099/acmi.0.001009.v3

**Published:** 2025-07-25

**Authors:** Zina Alfahl, Louise O’Connor, Dearbháile Morris, Terry J. Smith, Jean O'Dwyer, Paul D. Hynds, Martin Cormican, Liam P. Burke

**Affiliations:** 1 Antimicrobial Resistance and Microbial Ecology Group, School of Medicine, University of Galway, Galway, Ireland; 2 Centre for One Health, Ryan Institute, University of Galway, Galway, Ireland; 3 Molecular Diagnostics Research Group, College of Science & Engineering, University of Galway, Galway, Ireland; 4 School of Biological, Earth and Environmental Sciences, University College Cork, Cork, Ireland; 5 Irish Centre for Research in Applied Geosciences (iCRAG), University College Dublin, Dublin, Ireland; 6 Environmental Sustainability and Health Institute, Technological University Dublin, Dublin, Ireland

**Keywords:** filtration, quantitative real-time PCR, Shiga toxin-producing *Escherichia coli *(STEC), water contamination

## Abstract

Consequences of Shiga toxin-producing *Escherichia coli* (STEC) infection can range in severity from asymptomatic infection to haemolytic uraemic syndrome, renal failure and death. Groundwater-derived drinking water is an important route for STEC transmission. Detection of STEC in water is crucial for timely response and public health interventions; however, currently used culture-based methods are time-consuming and laborious. Therefore, there is a need for rapid methods that maintain high sensitivity and specificity [1]. We describe a novel, sensitive, enrichment-free water filtration method using a convenient sample volume (100 ml) to detect DNA markers of STEC serogroups and virulence factors within 6 h. Quantitative real-time PCR (qPCR) was used to detect and quantify the most common STEC infection-associated serogroups globally, O157 and O26. Real-time PCR was used to detect genetic determinants of STEC virulence (*stx1*, *stx2* and *eae* genes) and specific marker genes for the clinically relevant serogroups O111, O103, O145 and O104. Results showed that the novel method can detect as low as 5 c.f.u. ml^−1^ of STEC in water. The limit of detection for O157 and O26 qPCR assays was two and six copies, respectively. Groundwater and surface water samples (*n*=28) were collected and processed using the novel method. STEC O157 and O26 serogroups were detected in 23 out of 28 (82.1%) samples (mean 5.2×10^4^ copies/reaction) and 19 out of 28 (67.9%) samples (mean 7.83×10^4^ copies/reaction), respectively. Shiga toxin genes *stx1* or *stx2* were detected in 15 out of 28 (53.6%) and 9 out of 28 (32.1%) samples, respectively. The virulence factor intimin gene *eae* was detected in 24 out of 28 (85.7%) samples. STEC serogroups O111, O103, O145 and O104 were detected in 15 out of 28 (53.6%), 10 out of 28 (35.7%), 11 out of 28 (39.3%) and 15 out of 28 (53.6%) samples, respectively. This novel method reproducibly detects low copies of STEC in low-volume fresh water and has the potential to be used for the detection and quantification of waterborne bacterial pathogens.

## Data Summary

All data associated with this work are reported within the article and supplementary materials.

## Introduction

Shiga toxin-producing *Escherichia coli* (STEC) is an important zoonotic pathogen which is associated with a wide spectrum of diseases ranging in severity from asymptomatic carriage or moderate diarrhoea to haemorrhagic colitis. A potential complication is haemolytic uraemic syndrome, which can result in long-term renal and extra-renal sequelae and, in some cases, kidney failure and death [2]. The symptoms are caused by potent bacterial cytotoxins produced by STEC, known as Shiga toxins type 1 and type 2 (*stx1* and *stx2*), the former of which is closely related to the toxin produced by *Shigella dysenteriae* [3]. In 2022, there were 8,565 confirmed cases of STEC infection across 29 reporting EU/EEA countries, with an overall notification rate of 2.5 cases per 100,000 population and a fatality rate (where outcome was known) of 0.5%. STEC serogroups O157 (21.3%) and O26 (19.4%) were the most frequently reported amongst confirmed cases where serogroup information was available (42.2% of cases) [4]. The main reservoir of STEC is the gastrointestinal tract of ruminants, including cattle, sheep and other livestock [5]. The primary routes of transmission for STEC involve the transfer from animals to humans through direct contact with faeces or the ingestion of food or water contaminated with faecal matter [6]. Secondary person-to-person transmission is also a significant part of the epidemiology of STEC [7]. The ability of STEC to persist in diverse environmental conditions, coupled with its low infectious dose (10–100 cells), enables the bacterium to cause large-scale waterborne outbreaks [8].

In Ireland, a country that has reported the highest notification rate for STEC in Europe for most years in the last decade, around 17% of the population use private groundwater supplies for their daily needs. The overall quality of water in private supplies lags public supplies, attributed to limited source treatment, regulation and monitoring. Testing and treatment are entirely voluntary for household wells, and the cost and responsibility fall on the well owner [9]. In 2019 and 2022, exposure to water from a household well was reported in 27% of STEC infection cases [10]. Moreover, the incidence of confirmed sporadic STEC infection is significantly elevated in regions characterized by high reliance on such supplies for domestic drinking water [11].

The new Drinking Water Directive calls for a complete risk-based approach to water safety that considers more effectively the impact of climate change on water resources and focuses on assessing, monitoring and managing the main risks identified in the catchment areas and the supply system (Directive, 2023). Although STEC is undoubtedly amongst the main risks in an Irish context, the detection methods for STEC in water sources and supplies currently used during outbreak investigations are not fit for this purpose. Detection methods rely on membrane filtration, followed by enrichment and selective cultural isolation, which may include immunomagnetic separation of specific serogroups, with confirmation by serological and biochemical testing [12]. However, culture-based methods are labour-intensive and exhibit clear limits in analytical sensitivity for STEC detection, slow turnaround time (several days) and reduced ability to detect non-O157 and non-O26 serogroups. This creates a diagnostic gap since 50% or more of STEC infections may be caused by other STEC serogroups, which could result in incomplete surveillance of STEC infections [13]. Molecular identification of Shiga toxin or serogroup-associated genetic markers in filter enrichments according to the ISO/TS 13136 : 2012 real-time PCR method (ISO, 2019) is faster and identifies other prevalent serogroups but does not facilitate quantification.

Previous work in our group by Morris *et al*. [14] described the development and validation of a filtration-PCR protocol for capture and detection of STEC from environmental water [capture, amplify, extract (CapE)]. However, this method requires a large volume of water (30 l) and utilizes sample enrichment, thereby precluding enumeration [14]. The primary objective of the present study was to develop and assess a sensitive, enrichment-free, low-volume filtration and rapid lysis (ELR) molecular detection method to overcome these limitations, enabling effective and practical monitoring of STEC and quantification of key human infection-associated STEC serogroups O157 and O26 in environmental and drinking water samples.

## Methods

The ELR method does not involve lysing the cells; it focuses on direct detection without cell disruption. The ELR method comprises passing a 100 ml water sample through a 0.22 µm pore size, sterile Sterivex^™^ pressure-driven filter unit (Merck Millipore Ltd., Ireland). Following filtration, 400 µl of elution buffer (10 mM Tris-Cl, pH 8.5) is added to the filter unit, which is vortexed at a low speed for 10 min to elute the captured cells from the filter membrane. An aliquot (5 µl) of the eluate from the water sample is used as a template in real-time PCR or quantitative real-time PCR (qPCR) assays for qualitative or quantitative detection of STEC presence, respectively. The time from sample collection to result using the ELR method is less than 6 h.

### qPCR for the detection and quantification of VTEC O157 and O26 serogroups

qPCR was performed in triplicate using a standard curve on the Light Cycler 480 real-time PCR instrument (Roche, Switzerland). Two previously published PCR assays were optimized to quantify *E. coli* O157 and O26 copies and target the serogroup-specific genes *rfbE* and *wzx*, respectively [15]. The *rfbE* gene encodes the O157 antigen, essential for serotype identification, whilst the *wzx* gene is involved in the O-antigen polysaccharide transport, crucial for bacterial outer membrane structure. For each assay, an internal amplification control was used to provide assurance that the target gene was successfully amplified and detected (Data SA1 and Tables S1 and S2, available in the online Supplementary Material). A positive and a negative DNA control and no template control (NTC) were included in each qPCR run. Sample processing, reaction conditions, primers and TaqMan^™^ hydrolysis probes used are presented in Data SA1.

The limit of detection (LoD) of each assay was determined by preparing known concentrations of STEC O157 and O26 gBlocks^®^ DNA (Table S1). Eight replicates of concentrations equivalent to 10^8^, 10^7^, 10^6^, 10^5^, 10^4^, 10^3^, 10^2^, 10^1^, 8, 6, 4 and 2 copies in a 5 µl volume were tested in three independent experiments.

A standard curve was developed to quantify the copies per reaction for both STEC O157 and O26 serogroups according to The MIQE Guidelines: Minimum Information for Publication of Quantitative Real-Time PCR Experiments (Bustin *et al*., 2009). To generate an efficient standard curve, the standard (gBlock^®^ DNA) was serially diluted in triplicate starting from an initial concentration of 4×10^8^ copies per microlitre to 4×10^1^ copies per microlitre.

### Qualitative real-time PCR for the detection of STEC virulence factors and other clinically relevant serogroups

Multiplex PCR was performed in triplicate using the Light Cycler 480 real-time PCR instrument (Roche, Switzerland). Multiplex PCR assays for detection of STEC virulence (*eae*, *stx1* and *stx2*) and serogroup-specific genes (for O111, O103, O145 and O104 serogroups) were performed as previously described [1,16,18]. A positive and a negative DNA control and NTC were included in each PCR run. Sample processing, reaction conditions, primers and TaqMan hydrolysis probes used are presented in Data SA2.

### Validation of the ELR method for detection of STEC in spiked sterile water

This study adhered to the University of Galway’s biosafety regulations and guidelines for the handling of human pathogens. In accordance with the institution’s biosafety clearance, no culturing of the class III pathogen was carried out during the course of this work. To minimize the risk of infection, initial validation experiments were carried out by spiking 100 ml of sterile water with known concentrations of a non-toxigenic strain of intimin (*eae*)-positive, Shiga toxin (*stx1* and *stx2*)-negative *E. coli* ATCC 2216. The spiking concentrations of *E. coli* ATCC 2216 were 10^3^, 10^1^, 5 and 1 c.f.u. ml^−1^.

Spiked 100 ml water samples were passed through a 0.22 µm pore size, sterile Sterivex^™^ pressure-driven filter unit (Merck Millipore Ltd., Ireland). Then, 400 µl of elution buffer was added to the filter unit, and the filter unit was vortexed at 3,000 r.p.m. for 10 min to elute the captured cells from the filter membrane. An aliquot (5 µl) of the eluate from the spiked water samples was tested using a previously validated *eae* PCR assay [16]. All validation experiments outlined below were repeated in triplicate.

### Validation of the ELR method using water samples collected from rivers and wells

Water samples were collected from the Corrib catchment in the west of Ireland, County Galway. The Corrib catchment is considered a relatively high-risk catchment in terms of STEC risk factors with high laboratory-confirmed human infection rates [19]. Water samples (*n*=28) were collected from groundwater wells (*n*=13), rivers (*n*=12), turlough (seasonal lake) (*n*=2) and agricultural drain (*n*=1) sources to validate the new method.

Untreated (raw) groundwater samples were collected directly from the household tap, following disinfection of the tap with isopropanol and flushing for 90 s. Samples (1 l) were collected in sterile polypropylene containers and transported refrigerated back to the laboratory within 4 h. The samples were processed using the ELR method, and the eluate was tested using optimized qPCR assays for the most common STEC serogroups (O157 and O26) (Section 2.1). Eluates were also tested in triplicate for virulence (*eae*, *stx1* and *stx2*) and serogroup-specific genes *wbd1*, *wzx*, *ihp1* and *wzx* for serogroups O111, O103, O145 and O104 by multiplex real-time PCR assays (Qualitative real time PCR for the detection of STEC virulence factors and other clinically relevant serogroups).

### Comparison between the ELR method and the CapE method in freshwater

Water samples (*n*=10) were collected from groundwater wells (*n*=4), rivers (*n*=4) and agricultural drains (*n*=2) within the Corrib catchment and processed using the new ELR method (as described above) and the CapE method [14]. The performance of the two methods for STEC detection and quantification in environmental waters was compared, focusing on sample volume (100 ml vs 30 l), whether the CapE method’s enrichment step significantly increased the copies of STEC O157 and O26 serogroups, and time to result.

Both the final eluate (ELR method) and the extracted enrichment DNA (CapE method) were tested using qPCR assays to detect and enumerate STEC O157 and O26 serogroup target copies as described in qPCR for the detection and quantification of VTEC O157 and O26 serogroups. Quantitative results for each method were recorded (copies/reaction) and compared using a parametric matched-samples T-test.

### Statistics

Probit analysis was performed using Minitab 17 statistical software to determine the LoD for qPCR assays using hit rate analysis at 95% hit rate.

Data are presented as numbers and percentages for water samples which were positive for STEC virulence factors and serogroups and as means for normally distributed data. The assumptions of normality were tested using the Shapiro–Wilk test. Comparisons between independent data were made using an independent matched samples T-test for parametric data. All statistical analyses were performed using SPSS Statistics 28 (IBM) and defined by a threshold of *P*≤0.05 for statistical significance, as per convention.

## Results

### Determination of the LoD for STEC O157 and O26 real-time PCR assays

The LoD for the STEC O157 assay was two copies, whilst the LoD for the STEC O26 assay was six copies, indicating that both assays are highly sensitive in detecting low copies of STEC O157 and O26 serogroup target genes. Table S5 shows the hit rate analysis for the combined data from the three independent runs.

### Generation of a standard curve for STEC O157 and O26

**Fig. 1.**
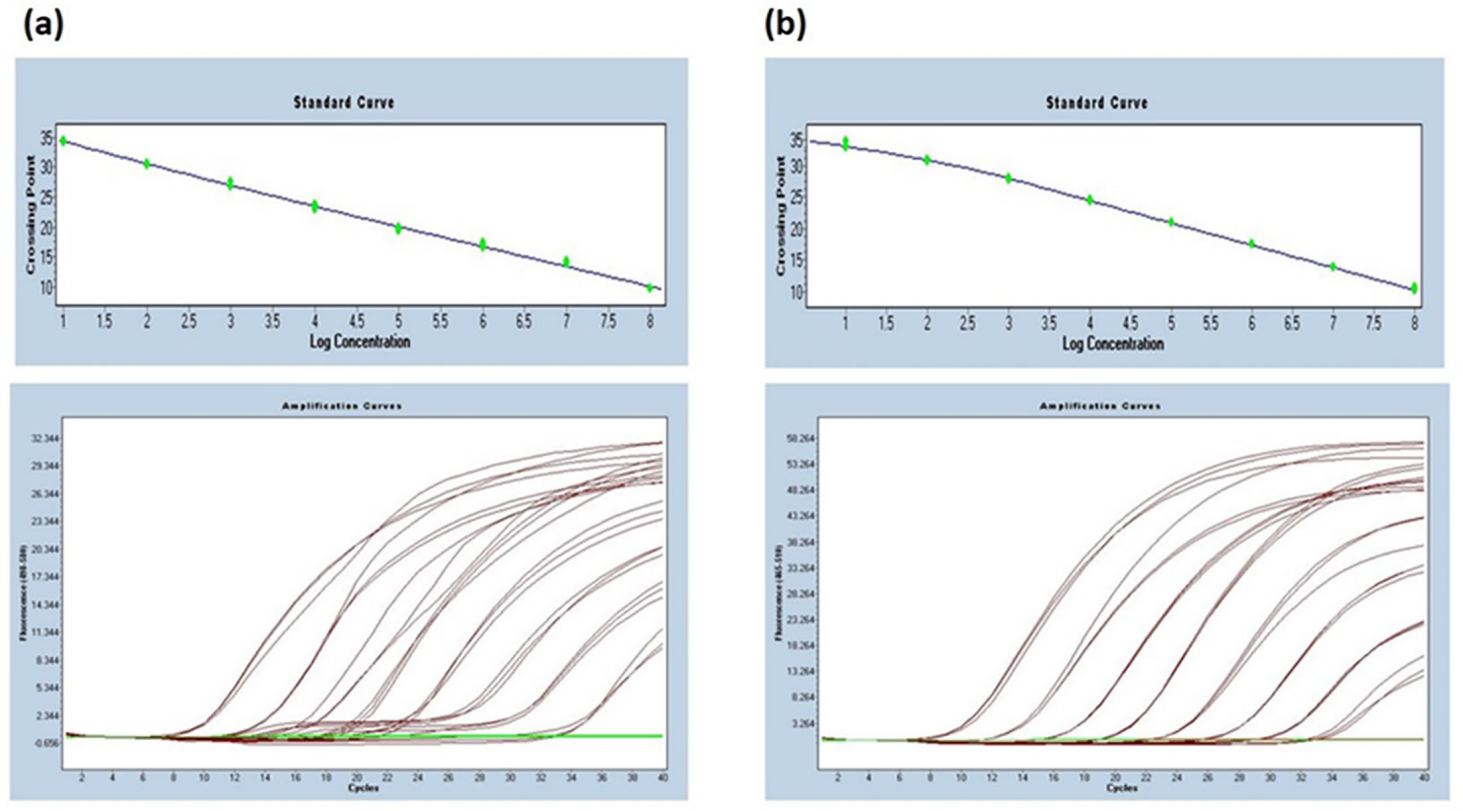
(a) STEC O157 standard curve (slope=−3.301, efficiency=100.9%) and (**b**) STEC O26 standard curve (slope=−3.520, efficiency=92.3%).

The efficiencies for both STEC O157 and O26 qPCR standard curves were 100.9% and 92.3%, respectively. Fig. 1 shows the standard curves and the amplification curves for both assays.

### Detection of virulence gene *eae* from the spiked sterile water using the ELR method

**Fig. 2.**
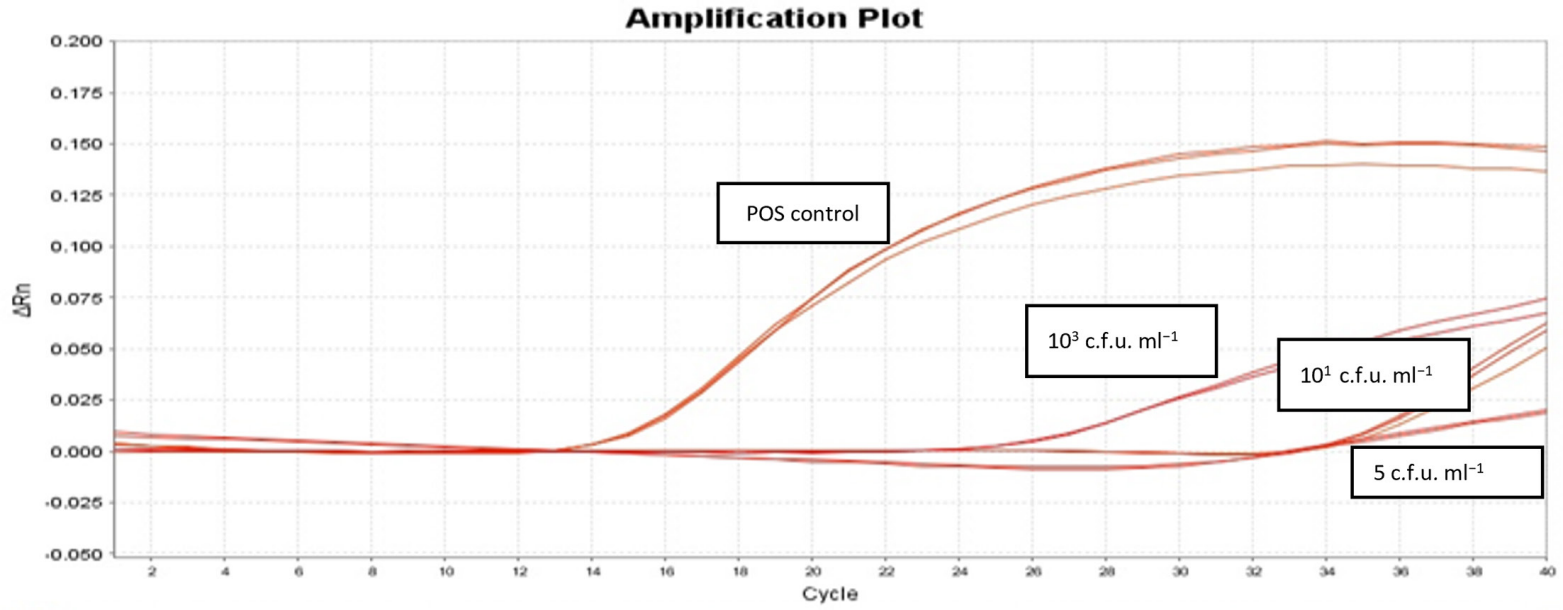
Amplification curves obtained when sterile water spiked with *E. coli* ATCC 2216 (103, 10^1^ and 5 c.f.u. ml^−1^) were tested for the *eae* gene. *E. coli* ATCC 2216 DNA (10^3^ copies) was used as a positive control.

The *eae* PCR assay was optimized with DNA extracted from *E. coli* ATCC 2216 with an LOD of one copy per microlitre equivalent achieved. The spiking concentrations tested of *E. coli* ATCC 2216 were as follows: 10^3^, 10^1^, 5 and 1 c.f.u. ml^−1^. The *eae* gene was consistently detected in three independent runs from water samples spiked with 10^3^, 10^1^ and 5 c.f.u. ml^−1^ indicating that the ELR method can detect as low as 5 c.f.u. ml^−1^ of STEC from water (Fig. 2).

### Validation of the ELR method for the detection of STEC serogroups and virulence genes in freshwater samples

The ELR method using qPCR facilitated quantitative detection of the STEC O157 serogroup gene *rfbE* in 23 out of 28 (82.1%) samples (mean 5.20×10^4^ copies/reaction). STEC O26 serogroup gene *wzx* was detected in 19 out of 28 (67.9%) samples (mean 7.83×10^4^ copies/reaction). Using PCR, Shiga toxin genes *stx1* or *stx2* were detected in 15 out of 28 (53.6%) and 9 out of 28 (32.1%) samples, respectively. The virulence factor intimin gene *eae* was detected in 24 out of 28 (85.7%) samples. Other STEC serogroup genes for O111, O103, O145 and O104 were detected in 15 out of 28 (53.6%), 10 out of 28 (35.7%), 11 out of 28 (39.3%) and 15 out of 28 (53.6%) samples, respectively (Table 1).

**Table 1.** Quantitative and qualitative detection of STEC -associated serogroups and virulence factors in surface and groundwater samples using the ELR method

green=gene detected; red=gene not detected. For copies/reaction, darker green indicates high copies (≥103 copies/reaction).

### Comparison between the ELR and CapE methods for detection of STEC O157 and O26 serogroups

STEC O157 and O26 were detected by both the ELR and CapE methods in 8 out of 10 (80%) and 5 out of 10 (50%) water samples, respectively (Table 2). A significant difference was observed in the average copies of STEC O157 detected between both methods (mean 1.66×10^1^ and 12.16×10^1^ copies; *P*=0.01, matched samples T-test; for the ELR and the CapE methods, respectively). No significant difference was observed in the average copy number of STEC O26 detected between both methods (mean 1.30×10^3^ and 7.33×10^3^ copies; *P*=0.22, matched samples T-test; for the ELR and the CapE methods, respectively). Furthermore, the time from sample collection to final result for the ELR and the CapE methods was 6 and 28 h, respectively.

**Table 2.** Detection and quantification of STEC O157 and O26 using the ELR and the CapE methods

green=detected; red=not detected.

## Discussion

Contamination of drinking water sources with STEC represents a major global public health concern, especially in regions where drinking water treatment is unregulated and represents a major transmission route for STEC infections and outbreaks [20,21]. Environmental water monitoring is vital to identify risks to drinking water source quality and potential sources of supply contamination. In outbreak situations, such testing can protect the public and facilitate decision-making on actions of water restrictions [22]. The recast Drinking Water Directive underscores the imperative for a robust assessment method capable of gauging the potential risks within a catchment area and its associated drinking water source, emphasizing the critical importance of safeguarding water quality for public health [23]. Therefore, rapid and sensitive screening methods are needed to detect and quantify pathogenic microbes in water, particularly pathogens such as STEC, where the infectious dose is low.

In this study, we developed an enrichment-free, rapid, sensitive method to detect STEC serogroups and virulence factors in low volumes of water. We optimized and validated the method to test 100 ml water samples, to align with the volume of water used for microbial quality testing by the European Commission’s Bathing Water Directive (European Commission, 2006) and recast Drinking Water Directive (Directive (EU) 2020/2184). The time from sample collection to results using this method is less than 6 h, which suggests its potential value as a tool for the quick identification of pathogens. Rapid detection and quantification of STEC in water are crucial to promptly identify and assess contamination levels, enabling swift intervention measures to prevent the spread of waterborne STEC, thereby mitigating potential public health risks. This method was demonstrated to be capable of detecting and quantifying each of the two most prevalent human infection-associated serogroups, O157 and O26, in a water sample.

A key advantage of the method is that it foregoes the need for sample enrichment, thereby providing unbiased hazard quantification data, suitable for use in risk assessment. A study by [24] also demonstrated that culture bias can arise during enrichment of samples containing multiple STEC serogroups. Specifically, they examined 14 STEC strains belonging to O157 and the so-called ‘big six’ non-O157 serogroups most commonly associated with human infection – O26, O45, O103, O111, O121 and O145. These serogroups are frequently targeted in public health monitoring due to their association with severe illness. When these strains were co-cultured in pairs and subjected to repeated enrichment over a 78 h period, a ≥2 log₁₀ difference in serogroup-specific gene copy numbers was observed in 12%, 38% and 52% of competitions at 30, 54 and 78 h, respectively. These percentages reflect an increasing prevalence of dominance by one strain over another as enrichment time progresses, indicating a time-dependent emergence of culture bias. Importantly, the study found that some strains consistently outcompeted others – regardless of serogroup – suggesting that strain-level characteristics such as Shiga toxin (*stx*) gene profile and individual fitness, rather than serogroup alone, dictate competitive success. Therefore, certain serogroups may be more difficult to isolate, not inherently, but due to being outcompeted during enrichment. This highlights the need to consider culture bias during method development and selection for hazard identification, especially when detecting mixed serogroup STEC in complex samples for risk assessment.

Comparing results of the enrichment-free ELR method with the previously developed CapE enrichment-based method further supports this serogroup-specific culture bias phenomenon, with a significant difference (*P*=0.01) noted in the copies of the STEC O157 serogroup, but not for STEC O26 copies. This finding has important implications for the interpretation of water quality results. The enrichment step used in the CapE method likely favours the growth or recovery of certain serogroups, such as O157, potentially leading to overrepresentation of these targets and underrepresentation of others in mixed populations. In contrast, the ELR method, which does not involve enrichment, captures a more immediate and possibly more representative snapshot of the microbial population present at the time of sampling. The discrepancy in O157 quantification between methods highlights the risk of overestimating the prevalence or concentration of certain serogroups when using enrichment-based protocols, which may influence public health risk assessments or regulatory responses. Conversely, the absence of a significant difference for O26 between methods suggests that some serogroups may be less affected by enrichment bias or may have different growth kinetics under enrichment conditions. These differences underscore the need for careful consideration of method selection when interpreting water quality data, especially for quantitative microbial risk assessment (QMRA). Enrichment-free methods like ELR may offer a more accurate estimation of contamination levels for real-time monitoring and risk management applications.

Although previous studies in the field suggest that membrane enrichment is necessary for increasing the sensitivity of the filtration method as well as for viability determinations [14,25,27], culture-dependent methods are time-consuming and labour-intensive. The ELR and qPCR methods demonstrated a high degree of sensitivity, detecting as low as two copies of STEC O157 and six copies of STEC O26 in spiked samples. This low LoD was further demonstrated when surface and groundwater samples were tested, as we were able to detect <10 copies. When compared with the enrichment-based CapE method, the ability to detect STEC was not impacted, despite the much lower (standard 100 ml) sample volume and shorter turnaround time. As the previously published CapE method was published by our group, the ELR method demonstrated that it achieves comparable detection levels without the need for large sample volumes or overnight culturing, thereby accelerating the identification of contaminated water sources.

A key limitation of this study is that the novel ELR method was only compared with the previously published CapE method, which is routinely used and validated within our laboratory. Whilst this provides a relevant internal benchmark, the study did not include comparisons with commercially available kits or standard culture-based methods for STEC detection due to resource constraints and biosafety restrictions, as the culture of pathogenic STEC strains was not feasible. Future work should therefore involve direct comparison of the ELR method with widely used commercial molecular assays and culture-based protocols, to further assess performance across different laboratory settings and evaluate broader diagnostic utility.

Another limitation of this study is the absence of basic water quality parameter data (e.g. pH, temperature and turbidity) for the collected samples. These environmental conditions can influence the survival, persistence and distribution of STEC in water sources and may also affect the performance of molecular detection methods. Whilst all samples were processed using the same protocol and no PCR inhibition was observed, indicating consistent assay performance, factors such as turbidity or organic load could potentially impact filtration efficiency or DNA recovery in more challenging water matrices. Future studies should incorporate the measurement of key water quality parameters to enable a more comprehensive assessment of how these variables influence the sensitivity and robustness of the ELR method across different environmental conditions.

For the purpose of this study, the qPCR assays targeted serogroup-associated genes for STEC O157 and O26, as they are currently the serogroups most frequently associated with human infection in Ireland [10]. Molecular detection of serogroup-specific and virulence factor genes is an appropriate indication of water contamination with STEC. Molecular methods are widely used for STEC detection in the clinical setting in Ireland, where some STEC cases are reported by the Health Protection Surveillance Centre based on PCR-positive results only [10,28, 29]. An additional benefit of our optimized qPCR assays for environmental detection is the ability to quantify the concentration of these serogroup markers in a sample. This enables monitoring the change in waterborne STEC concentration in any water source over time, including during specific events, such as extreme weather events or outbreaks. This will, in turn, inform effective risk management strategies for catchment areas, abstraction points and the entirety of the supply system, ensuring water safety throughout the journey from source to consumer delivery in accordance with the objectives of the recast Drinking Water Directive. One of the limitations associated with PCR is the inability to discriminate dead from live bacteria. However, detection of the serogroup and virulence gene targets in a water sample nonetheless indicates that contamination has occurred. Future studies can overcome this limitation by applying reverse transcriptase PCR that targets mRNA.

## Conclusions

We describe a rapid, sensitive, enrichment-free molecular method for the detection and quantification of low-level STEC contamination in waters. The method is economically feasible, as it utilizes low-cost materials and scalable processes that ensure affordability without compromising filtration performance. The method developed and validated in the current study can be applied to large-scale screening and epidemiological studies for rapid detection of STEC virulence genes and serogroups in water sources and supplies. Furthermore, this method can assist researchers to generate accurate quantitative data pertaining to both STEC O157 and O26 serogroups, for use in quantitative microbiological risk assessment (QMRA) models and to inform risk management approaches. Finally, the ELR method represents a potentially valuable tool for risk-based monitoring and could be applied for rapid detection and quantification of gene targets of interest in other waterborne bacterial pathogens, such as *Vibrio* spp. and *Shigella* spp.

## Supplementary material

10.1099/acmi.0.001009.v3 Uncited Supplementary Material 1.

## Abbreviations

CapE: capture, amplify, extract
ELR: enrichment-free, low-volume filtration and rapid lysis
LoD: limit of detection
NTC: no template control
PCR: polymerase chain reaction
QMRA: quantitative microbial risk assessment
qPCR: quantitative real-time PCR
STEC: Shiga toxin-producing *Escherichia coli*
