# Supplementary material for: A novel enrichment-free, low-volume filtration and rapid lysis (ELR) method in combination with real-time PCR for detection of Shiga toxin-producing Escherichia coli (STEC) in water
Source: Access Microbiol. 2025 Jul 25;7(7):001009.v3. doi: 10.1099/acmi.0.001009.v3 (PMC12451303; doi:10.1099/acmi.0.001009.v3)
Supplement: Uncited Supplementary Material 1. [file acmi-7-01009-s001.pdf]

A novel Enrichment-free, Low-volume filtration and Rapid lysis method (ELR) in combination with real-time PCR for detection of Shiga toxin-producing Escherichia coli (STEC) in water (Supplementary data)

Zina Alfahl [1,2+*], Louise O'Connor [2,3+], Dearbháile Morris [1,2], Terry J. Smith [3], Catherine Burgess [4], Jean O'Dwyer [5,6], Paul D. Hynds [6,7], Martin Cormican [1,2], Liam P. Burke [1,2]

[1] Antimicrobial Resistance and Microbial Ecology Group, School of Medicine, University of Galway, Galway, Ireland

[2] Centre for One Health, Ryan Institute, University of Galway, Galway, Ireland

[3] Molecular Diagnostics Research Group, College of Science & Engineering, University of Galway, Ireland

[4] Teagasc Food Research Centre, Ashtown, Dublin, Ireland

[5] School of Biological, Earth and Environmental Sciences, University College Cork, Cork, Ireland

[6] Irish Centre for Research in Applied Geosciences (iCRAG), University College Dublin, Ireland

[7] Environmental Sustainability and Health Institute, Technological University Dublin, Ireland

[+] These authors contributed equally

*Corresponding author at:

Antimicrobial Resistance and Microbial Ecology Group, School of Medicine, University of Galway, Galway, Ireland. Email address: zina.alfahl@universityofgalway.ie (Z.Alfahl)

A1. Quantitative real time PCR (qPCR)

Table S1 Internal amplification control (IAC), STEC O157 and STEC O26 gBlocks® sequences

Name	Sequence 5' – 3'
IAC gBlock®	TGA GGA AGA CTT ATT GGC TGA TAC CCA ACT TGG AAT GCT GTT TTG GGT TGG TCC TTA GAT GGT ACC AGT GCC AAT CCA GGG GAT ACA TTC ACA TTG AAT ATG CCA TGT GTG TTT AAA TAT ACT ACT TCA CAA ACA TCT GTT GAT TTA ACT GCC GAT GGT GTT AAA TAT GCT ACT TGT CAA TTT TAT TCT GGT GAA GAC TTA AAG ACC ACA ATG TAC CGG C
STEC O157 gBlock®	TCC ACA AGG AAA GTA AAG ATG TTT TTC ACA CTT ATT GGA TGG TCT CAA TTC TAA CTA GGA CCG CAG AGG AAA GAG AGG AAT TAA GGA ATC ACC TTG CAG ATA AAC TCA TCG AAA CAA GGC CAG TTT TTT ACC CTG TCC ACA CGA TGC CA
STEC O26 gBlock®	CGT CTC TGT ACG CGA CGG CAG AGA AAA TTA TTA AAT GTA TTC AGT CTA TAG CAA CCC CGT TAA ATC AAT ACT ATT TCA CGA GGT TGA TAA AGC AAC ATG AAT TGA AAT TAG AAC CAT ACA AAG TTG GAG AAT ATA AAA GCC TGC TAT ATG CAA GC

IAC: internal amplification control.

STEC O157 and O26 species specific primers and probes targeting *rfbE* and *wzx*, respectively were selected as previously described (Lawal et al., 2015). Table S2 shows the primers and probes used. The final reaction mix volume for each reaction contained 12.5 µL Taqman fast universal PCR Master Mix (Thermo Fisher Scientific, USA), 0.25 µL of 50 µM forward primer, 0.25 µL of 50 µM reverse primer, 0.1 µL of 50 µM probe, 0.25 µL of 50 µM of the IAC forward and reverse primers, 0.1 µL of 50 µM IAC probe, 1.3 µL PCR-grade water, 5 µL of 10^3 copies/µL IAC gBlock® DNA and 5 µL of target template eluate/DNA bringing the total reaction volume to 25 µL.

STEC O157 gBlock® DNA (10^3 copies/µL) was used as a positive control for the O157 assay and STEC O26 gBlock® DNA (10^3 copies/µL) was used as a positive control for O26 assay. No template control (NTC) was used as a negative control.

The cycling conditions were as follows: pre-incubation at 50 °C for 2 min and 95 °C for 20 s, followed by 40 cycles (95 °C for 3 s and 60 °C for 30 s). Data analysis was performed using real time Light Cycler PCR software.

Table S2 Primers and Probes for Internal amplification control (IAC), STEC O157 and O26 assays

	**Name**	**Sequence (5' to 3')**
IAC	Forward primer	TGAGGAAGACTTATTGGCTGATACCC
	Reverse [illegible]	GCCGGTACATTGTGGTCTTTAAGTC
	Probe	5Cy5-TCCTTAGATGGTACCAGTGCCAATC-3IAbRQSp
STEC O157 (*rfbE*)	Forward primer	TTTCACACTTATTGGATGGTCTCAA
	Reverse primer	CGATGAGTTTATCTGCAAGGTGAT
	Probe	JOE-AGGACCGCAGAGGAAAGAGAGGAATTAAGG-TAMRA
STEC O26 (*wzx*)	Forward primer	CGCGACGGCAGAGAAAATT
	Reverse primer	AGCAGGCTTTTATATTCTCCAACTTT
	Probe	FAM-CCCCGTTAAATCAATACTATTTCACGAGGTTGA-TAMRA

IAC: internal amplification control.

A2. Multiplex Real time PCR

Multiplex PCR assays for detection of virulence (*eae*, *vtx1* and *vtx2*) and serogroup-specific genes *wbd1*, *wzx*, *ihp1* and *wzx* for serogroups O111, O103, O145 and O104, respectively were performed as previously described (Bugarel et al., 2010; Nielsen and Andersen, 2003; Perelle et al., 2004; Perelle et al., 2005). Table S3 shows the primers and probes used.

gBlocks® DNA (10^3 copies/µL) of each target were used as a positive control for each target except for the *eae* target in which ATCC 2216 DNA was used a positive control (Table S4). No template control (NTC) was used as a negative control.

The cycling conditions were as follows: pre-incubation at 50 °C for 2 min and 95 °C for 20 s, followed by 40 cycles (95 °C for 3 s and 60 °C for 30 s). Data analysis was performed using the real time Light Cycler PCR software.

Table S3 Primers and Probes used for STEC multiplex PCR assays

**Serogroup/gene (reference)**	**Name**	**Sequence (5' to 3')**
*stx1** (Perelle et al., 2004)	Forward primer	TTT GTY ACT GTS ACA GCW GAA GCY TTA CG
	Reverse primer	CCC CAG TTC ARW GTR AGR TCM ACR TC
	Probe	FAM-CTG GAT GAT CTC AGT GGG CGT TCT TAT GTA A-BHQ1
*stx2** (Perelle et al., 2004)	Forward primer	TTT GTY ACT GTS ACA GCW GAA GCY TTA CG
	Reverse primer	CCC CAG TTC ARW GTR AGR TCM ACR TC
	Probe	CY5-TCG TCA GGC ACT GTC TGA AAC TGC TCC-BHQ2
*eae** (Nielsen and Andersen, 2003)	Forward primer	CAT TGA TCA GGA TTT TTC TGG TGA TA
	Reverse primer	CTC ATG CGG AAA TAG CCG TTA
	Probe	ROX-ATA GTC TCG CCA GTA TTC GCC ACC AAT ACC-BHQ2
STEC O111/*wbd*** (Perelle et al., 2004)	Forward primer	CGA GGC AAC ACA TTA TAT AGT GCT TT
	Reverse primer	TTT TTG AAT AGT TAT GAA CAT CTT GTT TAG C
	Probe	JOE-TTG AAT CTC CCA GAT GAT CAA CAT CGT GAA-BHQ1
STEC O103/*wzx**** (Perelle et al., 2005)	Forward primer	CAA GGT GAT TAC GAA AAT GCA TGT
	Reverse primer	GAA AAA AGC ACC CCC GTA CTT AT
	Probe	FAM-CAT AGC CTG TTG TTT TAT-MGB
STEC O145/*ihp*** (Perelle et al., 2004)	Forward primer	CGA TAA TAT TTA CCC CAC CAG TAC AG
	Reverse primer	GCC GCC GCA ATG CTT
	Probe	Cy5-CCG CCA TTC AGA ATG CAC ACA ATA TCG-BHQ2
STEC O104/*wzx*** (Bugarel et al., 2010)	Forward primer	TGTC GCG CAA AGA ATT TCA AC
	Reverse primer	AAA ATC CTT TAA ACT ATA CGC CC
	Probe	FAM-TTG GTT TTT TTG TAT TAG CAA TAA GTG GTG TC-BHQ1

*All 3 targets (*stx1*, *stx2*, *eae*) were optimised in a multiplex assay. ** All 3 targets (O111, O145, O104) were optimised in a multiplex assay. *** O103 assay was optimised as a singleplex assay.

Table S4 gBlocks® sequences for multiplex PCR assays positive controls

	**Sequence 5' – 3'**
stx1 gBlock®	TTTGTTACTGTGACAGCTGAAGCTTTACGTTTTCGGCAA<br>ATACAGAGGGGATTTCGTACAACACTGGATGATCTCAGT<br>GGGCGTTCTTATGTAATGACTGCTGAAGATGTTGATCTT<br>ACATTGAACTGGGG
stx2 gBlock®	GCATCCAGAGCAGTTCTGCGTTTTGTCACTGTCACAGCA<br>GAAGCCTTACGCTTCAGGCAGATACAGAGAGAATTTCGT<br>CAGGCACTGTCTGAAACTGCTCCTGTGTATACGATGACG<br>CCGGGAGACGTGGACCTCACTCTGAACTGGGGGCGAAT<br>CAGCAATGTGCTTCCGGAGTATCGGGGAGAGGATGGTG<br>TCAGAGTGGGGAGAATATCCTTTAATAATATATCAGCGA<br>TACTGGGGACTGTGGCCGTTATACTG
STEC O111 gBlock®	CGAGGCAACACATTATATAGTGCTTTGTTACACACTGAA<br>AGTTCTTAAAAGTGAATTGAATCTCCCAGATGATCAACAT<br>CGTGAATACCTTTGGCTAACTAAACACCAAATAAATGCTA<br>AACAAGATGTTCATAACTATTCAAAAAA
STEC O103 gBlock®	GAAAATCAAGGTGATTACGAAAATGCATGTTTTTATTGGC<br>GTGCATCAATTAAATTATCCTTCATAGCCTGTTGTTTTAT<br>TATAAGTACGGGGGTGCTTTTTTCCAGTAAAATATCAGA<br>ATACTTATTTT
STEC O145 gBlock®	CGATAATATTTACCCCACCAGTACAGCCGTACAGACTGA<br>CAGCACTGTCACCGATAAAAACCAGGCAGATCGCGACG<br>CGGCGCAAGATACCGCCATTCAGAATGCACACAATATC<br>GCAAGCATTGCGGCGGC
STEC O104 gBlock®	AGCAGATATCGCAGGTTTTATTGTCGCGCAAAGAATTTC<br>AACTTTACTTGGTTTTTTTGTATTAGCAATAAGTGGTGTC<br>GTTTCAAGTCAGGTTTCTAGGGCGTATAGTTTAAAGGAT<br>TTTACATCCATT

Table S5 Hit rate analysis for STEC O157 and O26 real-time PCR assays

	Target input genome copies	Number of replicates tested	Number of replicates detected in PCR assay
**STEC O157**	10^8	24	24
	10^7	24	24
	10^6	24	24
	10^5	24	24
	10^4	24	24
	10^3	24	24
	10^2	24	24
	10^1	24	24
	8	24	24
	6	24	24
	2	24	24
**STEC O26**	10^8	24	24
	10^7	24	24
	10^6	24	24
	10^5	24	24
	10^4	24	24
	10^3	24	24
	10^2	24	24
	10^1	24	24
	8	24	24
	6	24	21
	2	24	15

References

Bugarel M, Beutin L, Martin A, Gill A, Fach P. Micro-array for the identification of Shiga toxin-producing Escherichia coli (STEC) seropathotypes associated with Hemorrhagic Colitis and Hemolytic Uremic Syndrome in humans. Int J Food Microbiol 2010; 142: 318-29.

Lawal D, Burgess C, McCabe E, Whyte P, Duffy G. Development of a quantitative real time PCR assay to detect and enumerate Escherichia coli O157 and O26 serogroups in bovine recto-anal swabs. J Microbiol Methods 2015; 114: 9-15.

Nielsen EM, Andersen MT. Detection and characterization of verocytotoxin-producing Escherichia coli by automated 5' nuclease PCR assay. J Clin Microbiol 2003; 41: 2884-93.

Perelle S, Dilasser F, Grout J, Fach P. Detection by 5'-nuclease PCR of Shiga-toxin producing Escherichia coli O26, O55, O91, O103, O111, O113, O145 and O157:H7, associated with the world's most frequent clinical cases. Mol Cell Probes 2004; 18: 185-92.

Perelle S, Dilasser F, Grout J, Fach P. Detection of Escherichia coli serogroup O103 by real-time polymerase chain reaction. J Appl Microbiol 2005; 98: 1162-8.
